# Predictive value of different proportion of lesion HLA-G expression in colorectal cancer

**DOI:** 10.18632/oncotarget.22487

**Published:** 2017-11-18

**Authors:** Rui-Li Zhang, Xia Zhang, Shan-Shan Dong, Bing Hu, Qiu-Yue Han, Jian-Gang Zhang, Wen-Jun Zhou, Aifen Lin, Wei-Hua Yan

**Affiliations:** ^1^ Department of Gastrointestinal Surgery, Taizhou Hospital of Zhejiang Province, Wenzhou Medical University, Linhai, Zhejiang, P.R. China; ^2^ Human Tissue Bank, Wenzhou Medical University affiliated Taizhou Hospital of Zhejiang Province, Linhai, Zhejiang, P.R. China; ^3^ Medical Research Center, Wenzhou Medical University affiliated Taizhou Hospital of Zhejiang Province, Linhai, Zhejiang, P.R. China; ^4^ Department of Laboratory Medicine, Xianju People's Hospital, Xianju, Zhejiang, P.R. China

**Keywords:** HLA-G, colorectal cancer, prognosis

## Abstract

Differential expression of HLA-G has been observed among cancer types and tumors from individuals with the same type of cancer; however, its clinical significance is rather limited. In this study, expression and predictive relevance of HLA-G expression in 457 primary colorectal cancer (CRC, n_colon_ = 232, n_rectal_ = 225) patients was investigated. Data showed 70.7% (323/457) of the CRC were HLA-G expression when the above 5% (HLA-G_Low_) was considered as positive, which wasn't associated with patient survival (*p* = 0.109). However, HLA-G expression above 55% (HLA-G_High_) was associated with a worse prognosis of CRC patients (*p* = 0.042). Furthermore, a shorter survival was found for the female (*p* = 0.042) and elder (*p* = 0.037) patients whose HLA-G expression was above HLA-G_Low_ level. HLA-G expression above HLA-G_High_ level showed a worse prognosis for female (*p* = 0.013), elder (*p* = 0.023), colon cancer (*p* = 0.016), advanced tumor burden (T_3+4_, *p* = 0.018), regional lymph node status (N_1+2_, *p* = 0.044), and advanced clinical stage patients (AJCC _III+IV_, *p* = 0.037). In conclusion, our results demonstrated for the first time that combination of differential lesion HLA-G expression notably improved the value of traditional survival prediction for CRC patients.

## INTRODUCTION

Colorectal cancer (CRC) occurs in an estimated 376,300 new cases and 191,000 deaths in 2015 in China [1]. Tumor progression is dictated by the intimately continuous interaction between malignant cells and the tumor microenvironment such as immune effector molecules and immune regulatory factors, and infiltration of various immune cells [2, 3]. Unfortunately, tumor cells have developed various strategies to avoid recognition and destruction by the host immune milieus, and the resistant variants eventually results in cancer [4].

The immune suppressive molecule human leukocyte antigen G (HLA-G), is rarely observed in normal adult tissues. However, it is found frequently neoexpressed in most tumor cells as cell membrane-bound or soluble forms [5]. Amounts of *in vitro* and *in vivo* evidence showed HLA-G could directly interact with its receptors expressed on almost all types of immune cells or by the pathway of “trogocytosis”, revealing a broad immune inhibiting function on both innate and adaptive immune responses [6]. In clinical settings and animal models, earlier studies have demonstrated the aberrant neoexpressed HLA-G in various types of cancers was related to advanced tumor grade, more aggressive behavior and worse disease outcome [7].

To be mentioned, we recently found that levels of peripheral soluble HLA-G in CRC patients were strongly related to prognosis, and it could improve the prognostic value by traditional prognosticators [8]. Indeed, different proportion of lesion HLA-G expression has been found between different cancer types and also between tumors from individuals with the same type of cancer, and the prognostic significance of the different degree of HLA-G expression remains rather limited. With the combination of lesion HLA-G expression percentages, we also aim to evaluate whether it could improve the prognostic value of traditional clinical prognosticators.

In the current study, HLA-G expression in 457 primary colorectal cancer lesions was analyzed with immunohistochemistry, and the differential lesion HLA-G expression for prognostic stratification with traditional prognosticators was analyzed.

## RESULTS

### HLA-G expression in primary CRC lesions

**Figure 1:**
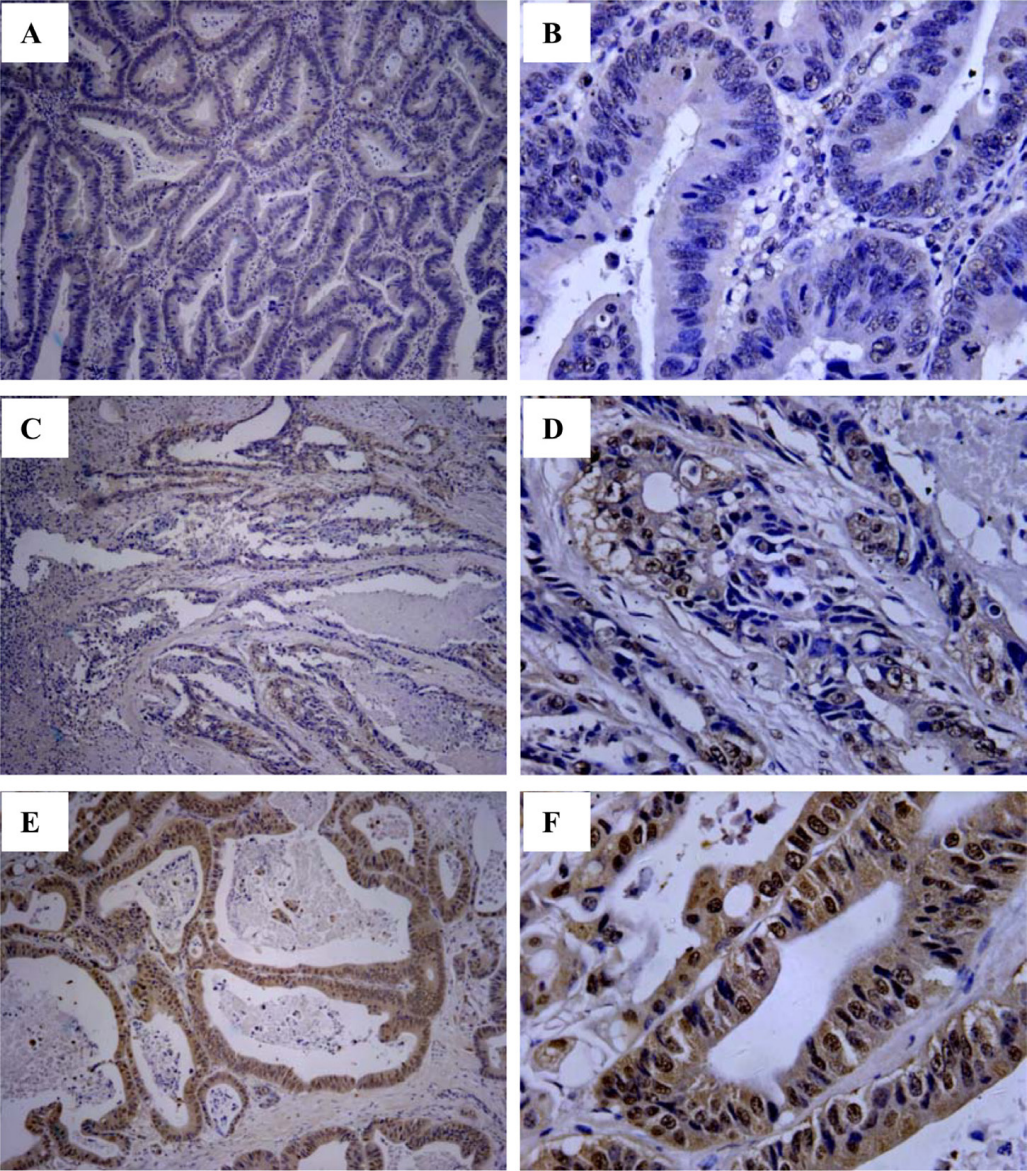
Immunohistochemistry analysis of HLA-G expression in primary CRC lesions Representative staining of negative (**A** and **B**), and positive expression (**C** and **D**; **E** and **F**) of HLA-G in CRC lesions. Original magnification: A, C, E (100×) and B, D, F (400×).

Different proportion of HLA-G expression was found and its expression in CRC lesions was from negative to 99% (Figure 1). Overall, 70.7% (323/457) of primary CRC samples were HLA-G positive, which was positive in 76.7% of the colon (178/232) and 64.4% of the rectal carcinoma lesions (145/225), respectively (Table 1).

**Table 1:** Association of lesion HLA-G expression with clinical parameters in CRC patients

Variables	No. of cases	HLA-G expression (5%, HLA-G_Low_)			HLA-G expression (55%, HLA-G_High_)		
		Neg.	Pos. (%)	p^*^	Neg.	Pos. (%)	p^*^
Histological type	457	134	323 (70.7)		158	299 (65.4)	
Colon carcinoma	232	54	178 (76.7)	0.004	64	168 (72.4)	0.001
Rectal carcinoma	225	80	145 (64.4)		94	131 (58.2)	
Gender							
Male	268	84	184 (68.7)	0.258	99	169 (62.7)	0.205
Female	189	50	139 (73.0)		59	130 (68.8)	
Age							
≤median (66 ys)	239	67	172 (72.0)	0.526	85	154 (64.4)	0.641
>median	218	67	151 (69.3)	73		145 (66.5)	
TNM stage							
Tumor status							
T_1+2_	113	28	85 (75.2)	0.128	34	79 (70.6)	0.147
T_3_	319	96	233 (69.9)		113	205 (64.3)	
T_4_	19	9	10 (52.6)		10	9 (47.4)	
Nodal status							
N_0_	242	68	174 (71.9)	0.826	83	159 (65.7)	0.997
N_1_	132	41	91 (68.9)		45	87 (65.9)	
N_2_	81	23	58 (71.6)		28	53 (65.4)	
Metastasis status							
M_0_	441	131	310 (70.3)	0.344	155	286 (64.9)	0.176
M_1_	16	3	13 (81.3)	3		13 (81.3)	
Disease stage							
I	90	24	66 (73.3)	0.695	29	61 (67.8)	0.522
II	149	44	105 (70.5)		54	95 (63.8)	
III	200	62	138 (69.0)		71	129 (64.5)	
IV	16	3	13 (81.3)		3	13 (81.3)	

^*^Comparison of HLA-G expression status between or among each variable using the Pearson chi-square test. TNM, lymph-node-metastasis and stage according to the TNM classification.

Kaplan–Meier survival analysis was performed to determine the minimum proportion of HLA-G expression which reaches statistic significance to patient survival. Data showed that, with the percentage at 5% as the cut-off value, HLA-G expression wasn't associated with the patient survival (*p* = 0.109), while HLA-G expression above 55% reached significantly to the patient survival (*p* = 0.042; Table 2). Base on this data, the different proportion of HLA-G expression in CRC lesions was divided into two groups as HLA-G_Low_ (cut-off = 5%) and HLA-G_High_ (cut-off = 55%) in this study.

**Table 2:** Log-rank Mantel–Cox analysis of clinical parameters in survival in CRC patients

Variables		No. Total	No. Events	Mean survival	95% CI	*p* value
Histological type	Colon	216	74	73.8	68.5–79.2	0.172
	Rectal	201	78	62.5	57.8–67.2	
Sex	Male	247	90	71.4	66.4–76.4	0.830
	Female	170	62	71.2	65.0–77.4	
Age	≤ 66 ys	215	75	73.7	68.4–79.0	0.219
	> 66 ys	202	77	68.7	63.0–74.4	
Tumor status	T_1+2_	102	21	83.4	76.5–90.3	<0.001
	T_3_	292	117	67.9	63.2–72.7	
	T_4_	18	11	54.8	36.5–73.1	
Nodal status	N_0_	217	50	83.5	78.7–88.2	<0.001
	N_1_	126	62	60.3	53.2–67.5	
	N_2_	72	40	51.5	41.5–61.5	
Metastasis status	M_0_	401	142	72.6	68.6–76.5	0.003
	M_1_	16	10	46.3	25.2–67.5	
Clinical stage	I	81	12	85.4	78.8–91.9	<0.001
	II	133	36	80.4	74.1–86.7	
	III	185	93	58.0	52.0–64.0	
	IV	16	10	46.3	25.2–67.5	
HLA-G_Low_	<5%	121	38	76.7	70.0–83.5	0.109
	>5%	296	114	68.5	64.0–73.2	
HLA-G_High_	<55%	144	46	77.4	71.4–83.4	0.042
	>55%	273	106	67.7	62.7–72.6	

Abbreviations: 95% CI = 95% confidence interval; TNM, lymph-node-metastasis and stage according to the TNM classification.

### Association between and HLA-G expression and clinical parameters

Data showed that (Table 1), a higher proportion of HLA-G expression was observed in colon carcinoma than that in rectal carcinoma lesions [HLA-G_Low_:76.7% (178/232) *vs.* 64.4% (145/225), *p* = 0.004; HLA-G_High_:72.4% (168/232) *vs.* 58.2% (131/225), *p* = 0.001], while no significance was found between the HLA-G expression and patient sex, age, primary tumor burden (T), regional lymph node status (N), distant metastases (M), and clinical disease stage. Also, no significant difference was observed for the relationship between the HLA-G expression and the clinical parameters either in colon or in rectal carcinoma patients (Supplementary Table 1).

### HLA-G status and clinical parameters to CRC patient survival

**Figure 2:**
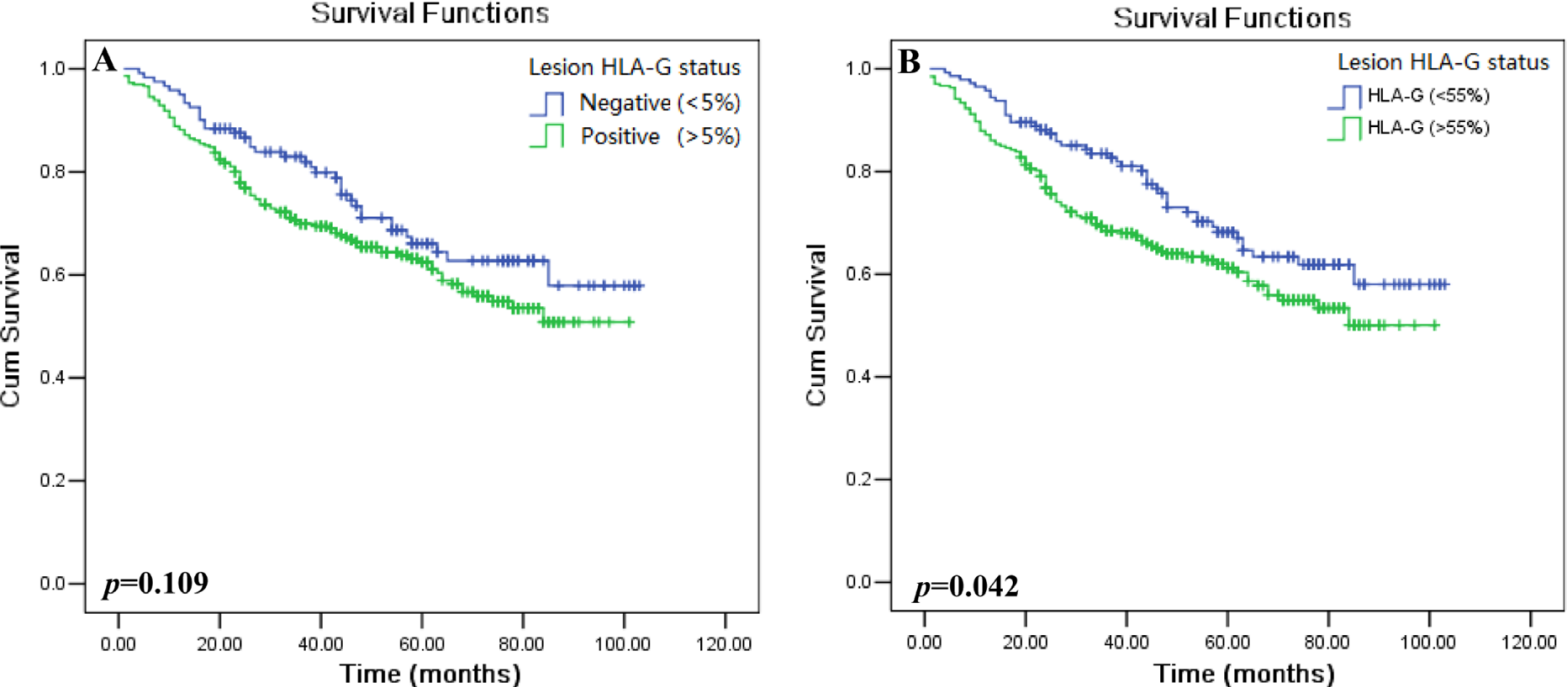
Kaplan–Meier survival analysis of HLA-G expression in CRC patients Comparison of the overall survival (**A**) between HLA-G negative and HLA-G positive patients with the cut-off = 5% (HLA-G_Low_; *p* = 0.109), and (**B**) between HLA-G negative and HLA-G positive patients with the cut-off = 55% (HLA-G_High_; *p* = 0.042).

Herein, HLA-G status and clinical parameters such as tumor histological type, patient sex, age, TNM categories, and clinical disease stage to the clinical outcome of CRC patients was evaluated. Data revealed that HLA-G_Low_ was not associated with patient prognosis (*p* = 0.109; Figure 2A). The mean survival for the HLA-G (<5%) and (>5%) in HLA-G_Low_ group was 76.7 months (*n* = 121; 95% CI: 70.0–83.5) and 68.5 months (*n* = 296; 95% CI: 64.0–73.2), respectively (Table 2). However, HLA-G_High_ was significantly related to the prognosis (*p* = 0.042, Figure 2B), where patients with HLA-G (>55%) had a worse outcome than patients with HLA-G (<55%) in HLA-G_High_ group. The mean survival for HLA-G (<55%) and (>55%) in HLA-G_High_ group was 77.4 months (*n* = 144; 95% CI: 71.4–83.4) and 67.7 months (*n* = 273; 95% CI: 62.7–72.6), respectively (Table 2).

**Figure 3:**
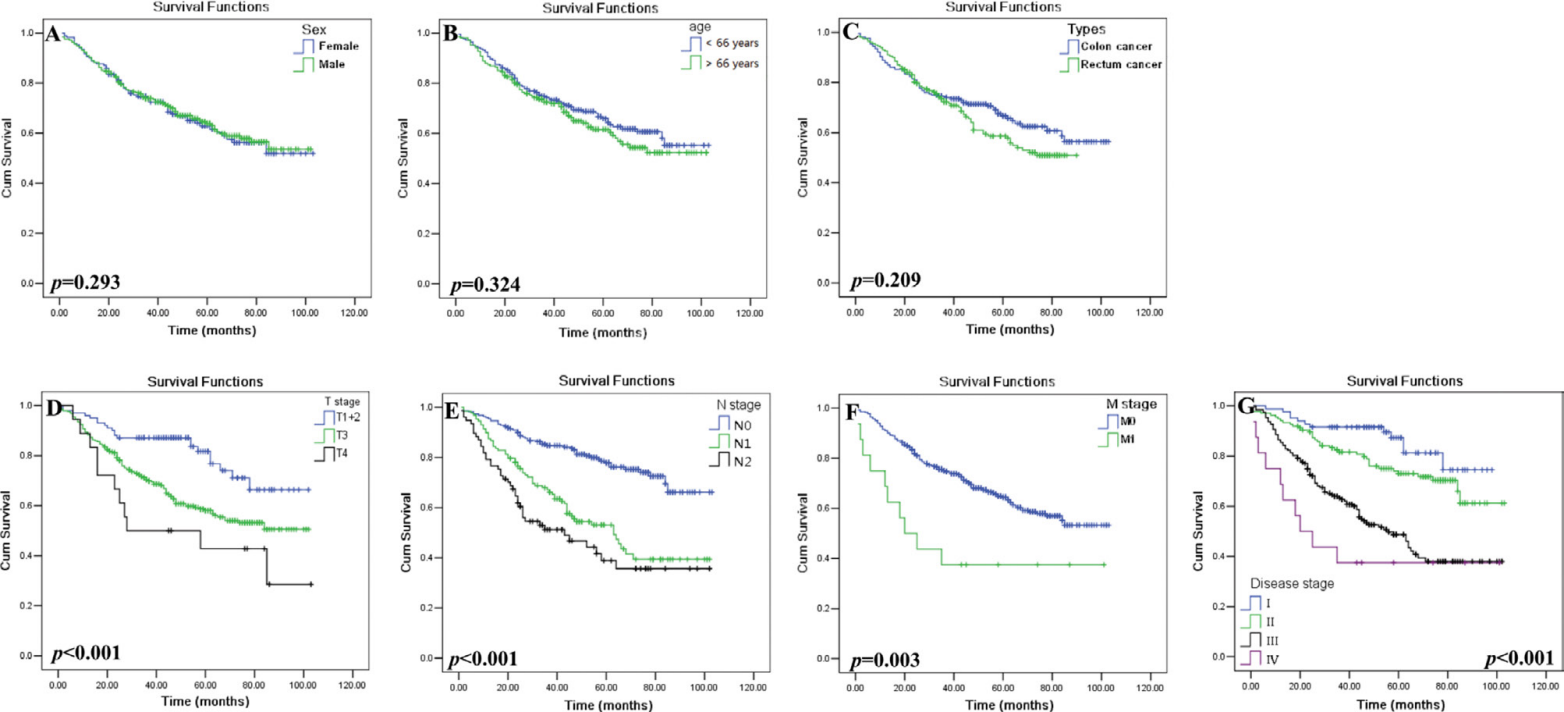
Kaplan–Meier survival analysis of clinical parameters in CRC patients Comparison of the overall survival between the (**A**) male (*n* = 247) and female (*n* = 170) patients (*p* = 0.293); (**B**) patients with age above (*n* = 202) and below (*n* = 215) the median of 66 years (*p* = 0.324); (**C**) tumor histological types of colon (*n* = 216) and rectal (*n* = 201) cancer (*p* = 0.209); (D) primary tumor status T_1+2_ (*n* = 102), T_3_ (*n* = 292) and T_4_ (*n* = 18; *p* < 0.001); (E) regional lymphnode status N_0_ (*n* = 217), N_1_ (*n* = 126) and N_2_ (*n* = 72; *p* < 0.001); (F) tumor metastasis status M_0_ (*n* = 401) and M_1_ (*n* = 16; *p* = 0.003); and (G) disease stages I (*n* = 81) , II (*n* = 133), III (*n* = 185) and IV (*n* = 16; *p* < 0.001) of CRC patients.

Among other clinicopathological variables, T, N, M categories and clinical disease stage was found significantly related to survival (Table 2, Figure 3). Patients with T_1+2_ [*n* = 102, mean: 83.4 months (95% CI: 76.5–90.3)] survived obviously longer than patients with T_3_ [*n* = 292; mean: 67.9 months (95% CI: 63.2–72.7)] or T_4_ [*n* = 18; mean: 54.8 months (95% CI: 36.5–73.1), *p* < 0.001]. Patients with N_0_ [*n* = 217, mean: 83.5 months (95% CI: 78.7–88.2)] had a better survival time than those with N_1_ [*n* = 126; mean: 60.3 months (95% CI: 53.2–67.5)] and N_2_ [*n* = 72; mean: 51.5 months (95% CI: 41.5–61.5), *p* < 0.001]. Patients with M_0_ (*n* = 401) had a better survival time than those with M_1_ [*n* = 16; mean: 72.6 months (95% CI: 68.6–76.5) *vs.* 46.3 months (95% CI: 25.2–67.5), *p* = 0.003]. Patients with stage I+II (*n* = 213) had a longer survival than those with stage III+IV [*n* = 201; mean: 83.7 months (95% CI: 79.2–88.7) *vs.* 57.2 months (95% CI: 51.4–63.0), *p* < 0.001]. The mean survival for stage I, I, III and IV was 85.4, 80.4, 58.0 and 46.3 months, respectively. However, CRC tumor histological type (*p* = 0.172), sex (*p* = 0.830) and patient age (*p* = 0.219) was not associated with patient survival.

Univariate analysis with Cox's proportional hazards model showed that clinical parameters including burden of primary tumor (T_3+4_ *vs.* T_1+2_, HR = 2.302, *p* < 0.001), regional lymph node status (N_1+2_ *vs.* N_0_, HR = 3.071, *p* < 0.001), distant metastases (M_1_ *vs.* M_0_, HR = 2.529, *p* = 0.005), and clinical disease stage (III + IV *vs.* I + II, HR = 3.162, *p* < 0.001) was significantly associated with a poor prognosis. For the HLA-G expression status, HLA-G_Low_ (>5% *vs.* <5%, HR = 1.348, *p* = 0.111) wasn't related to the prognosis, while HLA-G_High_ (>55% *vs.* <55%), HR = 1.428, *p* = 0.044) was associated with the prognosis (Table 3).

**Table 3:** Cox proportional hazards model analysis of variables in survival by HLA-G_Low_ or HLA-G_High_ expression in CRC patients

Variables	Categories	Univariate Analysis		Multivariate Analysis			
				HLA-G_Low_		HLA-G_High_	
		HR (95% CI)	*P*	HR (95% CI)	*P*	HR (95% CI)	*P*
Histological type	Rectal *vs.*Colon	1.248 (0.907–1.718)	0.174				
Sex	Female *vs.* Male	1.036 (0.750–1.432)	0.931				
Age (years)	>66 *vs.* ≤ 66	1.220 (0.887–1.678)	0.221				
Tumor status	T_3+4_ *vs.* T_1+2_	2.302 (1.451–3.654)	<0.001	1.679 (1.041–2.708)	0.034	1.694 (1.050–2.724)	0.031
Nodal status	N_1+2_ *vs.* N_0_	3.071 (2.184–4.318)	<0.001	1.416 (0.176–11.38)	0.744	1.428 (0.178–11.48)	0.737
Metastasis status	M_1_ *vs.*M_0_	2.529 (1.331–4.807)	0.005	1.501 (0.727–3.099)	0.273	1.458 (0.705–3.013)	0.309
Clinical stage	III+IV *vs.* I+II	3.162 (2.239–4.465)	<0.001	1.981 (0.238–16.48)	0.527	1.958 (0.235–16.29)	0.534
HLA-G_Low_ cut-off = 5%	Pos (>5%) *vs.* Neg (<5%)	1.348 (0.933–1.946)	0.111	1.423 (0.982–2.061)	0.062	/	/
HLA-G_High_ cut-off = 55%	Pos (>55%) *vs.* Neg (<55%)	1.428 (1.010–2.021)	0.044	/	/	1.481 (1.043–2.104)	0.028

Abbreviations: HR = hazard ratio; 95% CI = 95% confidence interval; TNM, lymph-node-metastasis and stage according to the TNM classification.

Multivariate analysis revealed that, HLA-G_High_ (HR = 1.481, *p* = 0.028) but not HLA-G_Low_ (HR = 1.423, *p* = 0.062), represented as an independent prognostic factor for CRC patients. Moreover, among traditional clinicopathological prognosticators, only the category of primary tumor burden (T) was found to be an independent prognostic factor when taking the status of HLA-G_Low_ (T_HR_ = 1.694, *p* = 0.031) and HLA-G_High_ (T_HR_ = 1.694, *p* = 0.031) as covariates respectively (Table 3).

### Significance of HLA-G status on the prognostic value of clinical parameters in CRC patients

Furthermore, we analyzed the prognostic significance of HLA-G status with stratification of clinical parameters in CRC patients. Briefly, The tumor histological type was stratified to colon and rectal carcinoma, patient sex to male and female, age to below and above the median age (66 years), categories T to T_1_, T_2_,T_3_ and T_4_; N to N_0_, N_1_ and N_2_; M status to M_0_ and M_1_, and clinical stage to I, II, III and IV, respectively.

**Figure 4:**
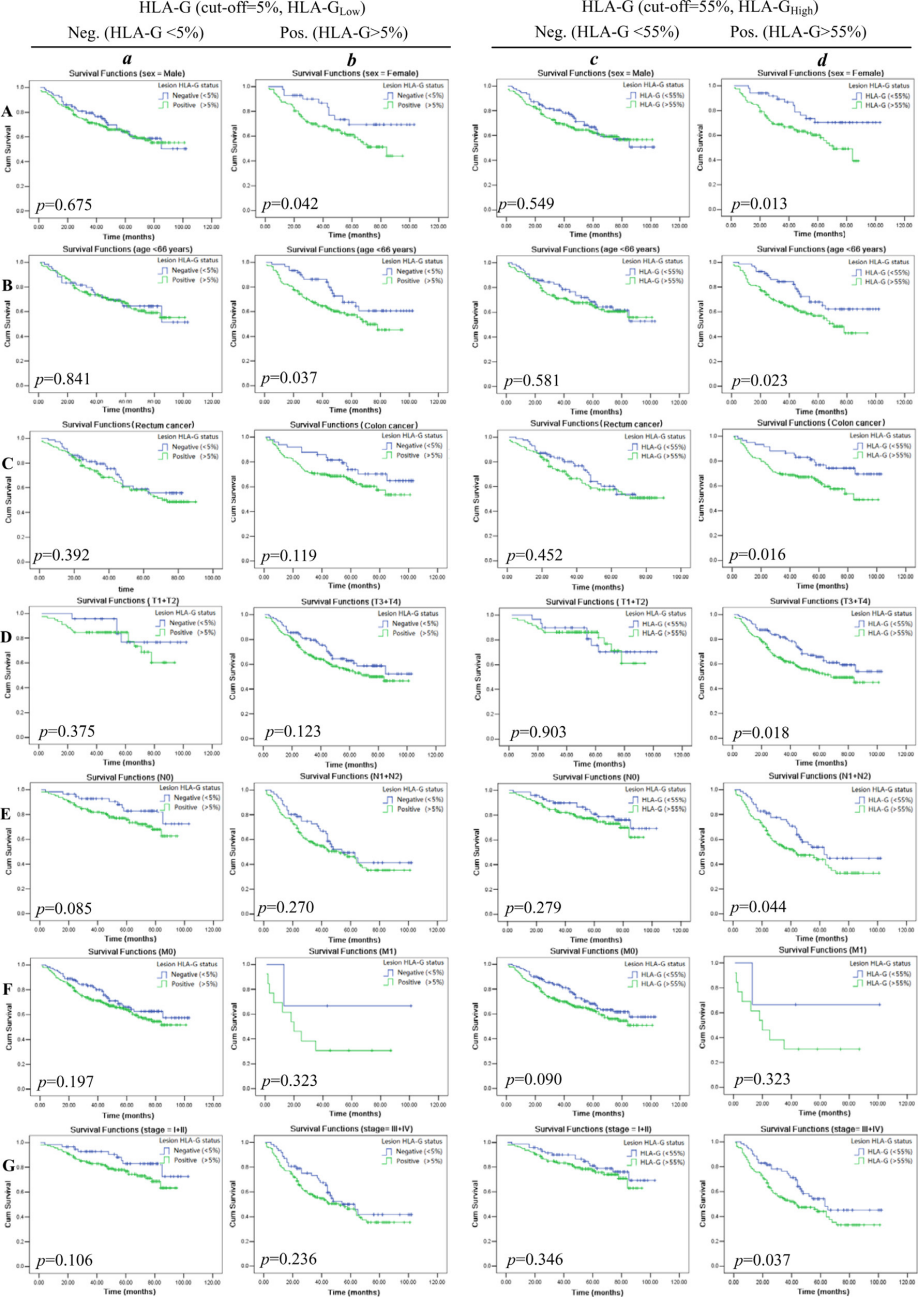
Kaplan–Meier survival analysis of stratified clinical parameters in survival by lesion HLA-G expression (HLA-G_Low;_ A~G-a and -b) and (HLA-G_High_; A~G-c and -d) in CRC patients, respectively Stratified clinical parameters (**A**) male and female patients; (**B**) patients with age above and below the median of 66 years; (**C**) tumor histological types of colon and rectal cancer; (**D**) primary tumor status T_1+2_ and T_3+4_; (**E**) regional lymph node status N_0_ and N_1+2_; (**F**) tumor metastasis status M_0_ and M_1_; and (**G**) disease stages I + II and III + IV of CRC patients.

Kaplan–Meier survival analysis revealed that both HLA-G_Low_ and HLA-G_High_ status could significantly affects the CRC patient survival when clinical parameters were stratified. To be noted, HLA-G_Low_ status (Table 4) was less powerful than the HLA-G_High_ status (Table 5) in affecting the patient survival between stratified clinical parameters. Data showed female patients whose HLA-G above the cut-off 5% (HLA-G_Low_) have a marked worse survival than those below the 5% level (mean: 63.5 months *vs.* 82.6 months, *p* = 0.042; Figure 4Ab). Similarly, the elder patients have worse survival with HLA-G above 5% (HLA-G_Low_) than those HLA-G expression was lower (mean: 61.3 months *vs.* 77.9 months, *p* = 0.037; Figure 4Bb). Furthermore, patients with HLA-G expression above the cut-off 55% (HLA-G_High_) have dramatically worse survival than those HLA-G expression was lower among the female (mean: 59.1 months *vs* 83.5 months, *p* = 0.013; Figure 4Ad), the elder patients (mean: 60.0 months *vs.* 78.2 months, *p* = 0.023; Figure 4Bd), and patients with colon carcinoma (mean: 68.1 months *vs* 84.8 months, *p* = 0.016; Figure 4Cd ), stage of T_3+4_ (mean: 62.5 months *vs.* 74.8 months, *p* = 0.018; Figure 4Dd), N_1+2_ (mean: 52.7 months *vs.* 65.4 months, *p* = 0.044; Figure 4Ed), and disease stage III+IV (mean: 52.7 months *vs.* 65.7 months, *p* = 0.037; Figure 4Gd ).

**Table 4:** Log-rank Mantel–Cox analysis of stratified variables in survival by lesion HLA-G expression with the cut-off value = 5% (HLA-G_Low_) in CRC patients

Variables	Stratified variables	HLA-G <5%				HLA-G >5%				
		No. Total	No. Events	Mean survival	95% CI	No. Total	No. Events	Mean survival	95% CI	*p* value
Histological type	Colon	49	14	82.4	73.1–91.8	167	60	70.2	64.0–76.4	0.094
	Rectal	72	24	60.7	54.0–67.5	129	54	61.1	55.2–67.0	
Sex	Male	79	28	72.8	64.2–81.3	168	62	70.3	64.3–76.4	0.114
	Female	42	10	82.6	71.8–93.4	128	52	63.5	56.9–70.0	
Age	≤66 ys	60	20	74.1	63.9–84.4	155	55	72.4	66.3–78.4	0.097
	>66 ys	61	18	77.9	68.9–87.0	144	59	61.3	54.8–67.7	
Tumor status	T_1+2_	23	4	89.8	79.2–100.4	78	17	75.9	68.5–83.3	0.060
	T_3_	87	28	75.0	66.9–83.1	205	89	64.5	58.8–70.1	
	T_4_	9	6	53.2	27.9–78.5	9	5	50.3	29.171.5	
Nodal status	N_0_	58	9	90.3	82.7–97.9	159	41	76.0	71.0–81.0	0.069
	N_1_	40	19	64.1	52.2–75.9	86	43	58.6	49.9–67.3	
	N_2_	21	10	57.6	38.4–76.8	51	30	43.4	34.3–52.6	
Metastasis status	M_0_	118	37	76.8	69.9–83.7	283	105	69.8	65.2–74.5	0.128
	M_1_	3	1	71.7	24.7–118.6	13	9	36.2	17.1–55.3	
Clinical stage	I	20	2	91.4	82.9–99.8	61	10	79.9	72.0–87.7	0.062
	II	38	7	87.1	76.7–97.6	95	29	73.2	66.6–79.9	
	III	59	28	62.0	51.8–72.3	126	65	56.1	48.8–63.4	
	IV	3	1	71.7	24.7–118.6	13	9	36.2	17.1–55.3	

Abbreviations: 95% CI = 95% confidence interval; TNM, lymph-node-metastasis and stage according to the TNM classification.

**Table 5:** Log-rank Mantel–Cox analysis of stratified variables in survival by lesion HLA-G expression with the cut-off value = 55% (HLA-G_High_) in CRC patients

Variables	Stratified variables	HLA-G <55%				HLA-G >55%				
		No. Total	No. Events	Mean survival	95% CI	No. Total	No. Events	Mean survival	95% CI	*p* value
Histological type	Colon	59	15	84.8	76.6–93.0	157	59	68.1	61.5–74.7	0.030
	Rectal	85	31	60.8	55.0–66.8	116	47	60.8	54.4–67.2	
Sex	Male	94	35	73.4	65.8–81.0	153	56	70.0	63.6–76.4	0.043
	Female	50	12	83.5	74.1–93.0	120	50	59.1	53.0–65.2	
Age	≤66 ys	77	26	75.9	67.4–84.3	138	49	71.6	65.0–78.2	0.045
	>66 ys	67	20	78.2	69.6–86.7	135	57	60.0	53.5–66.6	
Tumor status	T_1+2_	30	7	85.1	74.2–95.9	72	14	77.1	69.5–84.8	0.016
	T_3_	103	33	76.4	69.3–83.5	189	84	62.9	56.9–68.9	
	T_4_	10	6	58.9	34.0–83.8	8	5	45.1	23.8–66.5	
Nodal status	N_0_	73	15	87.5	80.5–94.4	144	35	75.7	70.4–81.0	0.027
	N_1_	44	20	66.0	54.9–77.1	82	42	57.6	48.7–66.6	
	N_2_	25	11	63.3	46.5–80.1	47	29	40.0	30.5–49.4	
Metastasis status	M_0_	141	45	77.5	71.5–83.6	260	97	69.0–	64.0–74.0	0.057
	M_1_	3	1	71.7	24.7–118.6	13	9	36.2	17.1–55.3	
Clinical stage	I	25	5	84.4	73.7–94.9	56	7	82.3	74.3–90.3	0.022
	II	48	10	86.9	78.0–95.8	85	26	69.9	63.1–76.8	
	III	67	30	65.4	56.0–74.8	118	63	53.9	46.2–61.4	
	IV	3	1	71.6	24.7–118.6	13	9	36.2	9.8–17.1	

Abbreviations: 95% CI = 95% confidence interval; TNM, lymph-node-metastasis and stage according to the TNM classification.

## DISCUSSION

In some cancers, patients within the same TNM stage but their clinical outcomes varied significantly, such as rapid disease progression and cancer-related death with early stage; however, advanced stage cancer can remain stable for years in some patients is often seen [9]. The major reason for the limited predictive power of the traditional staging system is that relies only on the tumor cell characteristics but ignores the effects of the host immune response against the cancers [10]. Indeed, increasing evidence has documented host immune contexture in tumor microenvironment play a critical role in the prediction of prognosis, such as the prognostic value of CD3+, CD8+ and CD45RO+ T cell infiltration in CRCs [11, 12].

Among various factors of host immune contexture in tumor immunology, induction of an immunotolerant HLA-G expression by tumor cells has been observed in numerous tumoral tissues [13]. HLA-G have multiple immune inhibitory effects such as impairing T cell functions by inhibiting of proliferation and cytotoxicity, induction of apoptosis and expansion of regulatory T cells [5]. Moreover, HLA-G expression was found inversely related to the frequency of intratumoral lymphocyte infiltration such as CD8+ T cells or the numbers of peripheral activated T cells (CD8+CD28+ T cells) [14, 15]. Previous studies showed that tumor HLA-G expression was associated with advanced stage or worse clinical outcome and its potential as a prognostic biomarker has been intensively investigated [5]. In CRC patients, Guo *et al*. [16] evidenced HLA-G was significantly related to the overall survival of CRC patients and it could be an independent prognosticator, similar findings were obtained in a study by Ye *et al.* [17]. Recently, Kuppen and colleagues [18, 19] found that absence of HLA-G expression indicated a better survival for colon cancer patients and weak expression of HLA-G revealed a worse survival in rectal cancer patients.

Our findings in the current study revealed that different proportion of HLA-G expression in CRC lesions is of significance to the patient survival or prognosis. Analogously, in our recent study, patients with higher levels of peripheral sHLA-G had a significantly worse survival than those with lower levels, and sHLA-G was considered as an independent prognostic factor for CRC patients. Moreover, with stratification of clinical parameters in survival by sHLA-G_low_ and sHLA-G_high_, could improve the prognostic power by traditional prognosticators in CRC patients [8]. Our previous *in vitro* studies had reported that inhibition of NK cytolysis is dependent on the proportion of HLA-G expression. In these studies, data showed that the power of HLA-G in NK cell cytotoxicity was dependent on the level of both HLA-G1 and HLA-G5 expression, and HLA-G1 and HLA-G5 have an additive effect on the NK cell cytolysis suppression [20]. Importantly, a significant inhibition would be reached when the HLA-G expression was more than 60% [21]. Thus, it's reasonable to speculate that the different proportion of HLA-G expression in tumor lesions could influence disease progression and clinical outcome.

In consistent with our mentioned above findings, CRC lesion HLA-G expression status also could significantly affect the CRC patient survival with the stratified clinical parameters. Applying HLA-G expression above the cut-off level at 5% (HLA-G_Low_) as positive which were commonly used in previous studies, HLA-G_Low_ was not significantly related to the CRC patient survival; however, when HLA-G expression above the level of 55% (HLA-G_High_), HLA-G_High_ reaches a statistic significance point to a worse survival, which echoes a study by Kirana *et al*. [22] that high, but not negative and moderate local HLA-G expression was closely linked to the CRC patient survival.

Degree of HLA-G expression could be influenced by multiple factors and mechanisms are complex. HLA-G genetic variation such as polymorphisms in 5′upstream regulatory region and 3′untranslated regions affects the affinity of targeted gene for transcriptional or post-transcriptional factors [23], epigenetic pathways through DNA methylation and histone modifications [24], post-transcriptional mechanisms by and microRNAs [25], as well as environmental factors including various cytokines, growth factors and hormones [26]. However, mechanisms underlying the individual or tumor-specific expression of HLA-G are required to be explored. Moreover, different proportion of lesion HLA-G expression has been found between different cancer types, between tumors from individuals with the same type of cancer, and even between the different intra-tumor areas within a single sample. In this context, Rouas-Freiss *et al*. recently reported that HLA-G and other immune molecules such as PD1/PDL1,ILT2/4 were heterogeneous expressed in the various areas of the same tumor [27]. These findings further highlights more detail and comprehensive evaluation of the intra- and inter- heterogeneity of HLA-G expression is necessary for explore the clinical significance the HLA-G in tumor biology.

Finally, our data showed that HLA-G_High_ status was powerful in affecting the patient survival when clinical parameter was stratified. Among female patients, the elder patients, colon carcinoma patients, stage of T_3+4_, N_1+2_, and stage III+IV whose HLA-G expression above the cut-off 55% (HLA-G_High_) have dramatically poor survival than those with lower HLA-G expression. In serous ovarian carcinoma, a study by Andersson *et al*. [14] revealed that HLA-G expression was correlated to a significant worse prognosis in patients with the genotype HLA-A*02. These data indicated that HLA-G expression status together with other clinical parameters, tumor microenvironment factors is possible to discriminate subpopulations and identify patients with even worse prognosis.

Our study demonstrated that different proportion of HLA-G expression in CRC patients influence the patient survival and a combination of HLA-G expression status with traditional clinical risk factors could refine the prediction of specific clinical outcomes of the subpopulations of CRC patients. Taken together, lesion HLA-G expression percentages in patients with CRC could be another prognostic factor which contributes an additional significance to the classical cancer TNM classification system.

## MATERIALS AND METHODS

### Colorectal cancer patients

Consecutive 457 colorectal cancer lesions including 232 primary colon carcinomas and 225 rectal carcinomas were obtained from patients diagnosed at Taizhou Hospital of Zhejiang Province from November 9th, 2004 to September 12th, 2012. Biosamples were provided by the Tissue Bank of Taizhou Hospital of Zhejiang Province, National Human Genetic Resources Sharing Service Platform (2005DKA21300).

Patient data including age (median: 66 years; range:26 years-90 years), gender (n_male_ = 268; n_female_ = 189), date of initial diagnosis and surgical operation, TNM status including size and extent of primary tumor (T), regional lymph node status (N) and distant metastases (M), and clinical disease stage were documented. The clinical stage classification follows the 7th TNM staging system by UICC and the AJCC [28]. All specimens were pathologically confirmed. All samples were anonymously analyzed in accordance with the Declaration of Helsinki. The study protocol was approved by the Ethical Board of the Taizhou Hospital of Zhejiang Province and a written informed consent was obtained from all patients.

Clinical stage of 455 cases was available among 457 cases. There were 90, 149, 200 and 16 patients with clinical stage I, II, III and IV, respectively. Of them, 417 patients were followed till the last follow-up at May 3rd, 2012. Overall survival was defined from the surgical operation date to the patient death (event) or last follow-up (censored) with the median follow-up of 46.5 months (range: 1–103 months), and 152 cancer-related deaths were occurred which includes 12 (15.0%) stage I, 36 (27.1%) stage II, 93 (50.3%) stage III and 10 (62.5%) stage IV patients, respectively.

### Immunohistochemistry and staining evaluation

The four-micrometer paraffin-embedded sections were dewaxed and rehydrated, and incubated overnight at 4°C with the anti-HLA-G mAb 4H84 (1:500, Exbio, Prague, Czech Republic), then thoroughly washed. Finally, sections were stained with Dako EnVison kit (Dako, Glostrup, Denmark).

CRC lesion HLA-G expression was evaluated by two reviewers who have no knowledge of to clinical information for these patients. The percentage of positive cells was based on the presence or absence of HLA-G staining, irrespective of staining intensity. Percentage of HLA-G positive tumor cells was evaluated by each reviewer, and the average was calculated. A sample was considered as positive when HLA-G positive CRC cells was >5% [29].

### Statistical analysis

SPSS 13.0 software (SPSS, Inc., Chicago, IL, USA) was used for statistical analysis. Categorical data were analyzed with Pearson chi-square test. Kaplan–Meier and log-rank test was performed for survival analysis. Relationship between the survival and variables were evaluated with the Cox regression method. Significant difference was considered as *p* < 0.05 (two-tailed).

## SUPPLEMENTARY MATERIALS FIGURES AND TABLES


