# Supplementary material for: Predictive value of different proportion of lesion HLA-G expression in colorectal cancer
Source: Oncotarget. 2017 Nov 18;8(64):107441–51. doi: 10.18632/oncotarget.22487 (PMC5746078; doi:10.18632/oncotarget.22487)
Supplement: Supplementary file 1 [file oncotarget-08-107441-s001.pdf]

Predictive value of different proportion of lesion HLA-G expression in colorectal cancer

SUPPLEMENTARY MATERIALS

Supplementary Table 1: Association of lesion HLA-G expression with clinical parameters in colon and rectal carcinoma patients

Variables	Colon Ca.	HLA-G (5%, HLA-G$_{Low}$)			HLA-G (55%, HLA-G$_{High}$)			Rectal Ca.	HLA-G (5%, HLA-G$_{Low}$)			HLA-G (55%, HLA-G$_{High}$)		
		Neg.	Pos. (%)	p^*	Neg.	Pos. (%)	p^*		Neg.	Pos. (%)	p^*	Neg.	Pos. (%)	p^*
No.	232	54	178 (76.7)		64	168 (72.4)		225	80	145 (64.4)		94	131 (58.2)	
Sex														
Male	130	34	96 (73.8)	0.275	40	90 (69.2)	0.239	138	50	88 (63.8)	0.790	59	79 (57.2)	0.709
Female	102	20	82 (80.4)		24	78 (76.5)		87	30	57 (65.5)		35	52 (59.8)	
Age														
≤median	120	26	94 (78.3)	0.641	34	86 (69.2)	0.883	114	40	74 (64.9)	0.882	50	64 (56.1)	0.521
>median	112	28	84 (75.0)		30	82 (73.2)		111	40	71 (64.0)		44	67 (60.4)	
Tumor status														
T_{1+2}	81	16	65 (80.2)	0.321	19	62 (76.5)	0.227	32	12	20 (62.5)	0.604	15	17 (53.1)	0.391
T_3	138	33	105 (76.1)		39	99 (71.7)		181	63	118 (65.2)		74	107 (57.5)	
T_4	13	5	8 (61.5)		6	7 (53.8)		6	4	2 (33.3)		4	2 (33.3)	
Nodal status														
N_0	133	30	103 (77.4)	0.925	38	95 (71.4)	0.923	109	38	71 (65.1)	0.692	45	64 (58.7)	0.999
N_1	64	15	49 (71.8)		17	47 (73.4)		68	26	42 (61.8)		28	40 (58.8)	
N_2	35	9	26 (74.3)		9	26 (74.3)		46	14	32 (69.7)		19	27 (58.7)	
Metastasis														
M_0	221	53	168 (76.0)	0.254	63	158 (71.5)	0.160	220	78	142 (64.5)	0.834	92	128 (58.2)	0.935
M_1	11	1	10 (90.9)		1	10 (90.9)		5	2	3 (60.0)		2	3 (60.0)	
Disease stage														
I	64	14	50 (78.1)	0.663	16	48 (75.0)	0.388	26	10	16 (61.5)	0.976	13	13 (50.0)	0.805
II	67	16	51 (76.1)		22	45 (67.2)		82	28	54 (65.9)		32	50 (61.0)	
III	90	23	67 (74.4)		25	65 (72.2)		110	39	71 (64.5)		46	64 (58.2)	
IV	11	1	10 (90.9)		1	10 (90.9)		5	2	3 (60.0)		2	3 (60.0)	

*Comparison of HLA-G expression status between or among each variable using the Pearson chi-square test.
